# Long-term health outcomes of people without celiac disease avoiding gluten consumption: a 25-year prospective cohort study

**DOI:** 10.1038/s41430-025-01641-x

**Published:** 2025-07-08

**Authors:** Eeva Salmela, Kurppa Kalle, Katri Lindfors, Päivi Saavalainen, Heini Huhtala, Katri Kaukinen, Juha Taavela

**Affiliations:** 1 Celiac Disease Research Center, Faculty of Medicine and Health Technology, Tampere University and Tampere University Hospital, Tampere, Finland; 2 Tampere Center for Child, Adolescent, and Maternal Health Research, Tampere University, Tampere, Finland; Department of Pediatrics, Tampere University Hospital, Wellbeing Services County of Pirkanmaa, Tampere, Finland; 3 University Consortium of Seinäjoki, Seinäjoki, Finland; 4 Department of Medical and Clinical Genetics, University of Helsinki, Helsinki, Finland; 5 Faculty of Social Sciences, Tampere University, Tampere, Finland; 6 Department of Internal Medicine, Tampere University Hospital, Wellbeing Services County of Pirkanmaa, Tampere, Finland; 7 Department of Gastroenterology and Alimentary Tract Surgery, Tampere University Hospital, Wellbeing Services County of Pirkanmaa, Tampere, Finland

**Keywords:** Functional gastrointestinal disorders, Intestinal diseases, Nutrition disorders

## Abstract

**Background:**

Self-reported abdominal symptoms after consuming gluten-containing cereals in individuals without celiac disease (CeD) are common. The long-term outcomes of these individuals are unknown.

**Methods:**

Seventy-six adults experiencing symptoms from gluten-containing cereals underwent exclusion of CeD and wheat allergy in 1995–1997 and were thus advised to revert to a normal gluten-containing diet. These individuals were invited to a comprehensive health examination, including measurement of CeD antibodies and symptoms and assessment of quality of life using the Gastrointestinal Symptom Rating Scale (GSRS) and Psychological General Well-Being Index (PGWB). Healthy individuals (*n* = 160) and untreated CeD patients (*n* = 128) served as controls.

**Results:**

Altogether, 28 individuals participated, half of whom were still avoiding gluten-containing cereals. None had acquired a diagnosis of any gastrointestinal disease, and all had negative CeD serology. The entire study group presented with significantly higher GSRS total (participants 2.8, 95% confidence interval 2.5–3.1 vs. controls 1.8, 1.7–1.9; *p* < 0.001) and other sub-scores than healthy controls, and higher total (vs. patients 2.5, 2.3–2.6; *p* = 0.041) and constipation scores than untreated CeD patients. Additionally, the group had worse PGWB total (participants 92.1, 84.9–99.4 vs. controls 105.3, 102.5–108.7; *p* = 0.002) and anxiety, self-control, general health, and vitality sub-cores than healthy controls, as well as self-control, general health, and vitality scores than untreated CeD patients. Twelve participants fulfilled the criteria for irritable bowel syndrome.

**Conclusions:**

None of the participants had developed CeD or been diagnosed with gastrointestinal disease for 25 years. They reported more gastrointestinal symptoms and a poorer quality of life, even when compared to untreated CeD patients.

## Introduction

Non-celiac gluten sensitivity (NCGS) is defined as the presence of gluten-induced symptoms without evidence of celiac disease (CeD) or wheat allergy [1]. Patients may experience gastrointestinal symptoms such as bloating, diarrhea, and abdominal pain, as well as various extraintestinal symptoms such as myalgia, tiredness, and a foggy mind. The main diagnostic feature is a beneficial response to dietary gluten avoidance. A 15-week placebo-controlled gluten challenge trial, including a 3-week crossover trial, has been suggested as a confirmatory test for NCGS; however, this is challenging to implement, and diagnostic performance is unclear [2]. Consequently, most of the diagnoses are based on self-report, making it difficult to estimate the causal role of gluten in causing the symptoms and to provide research-based guidelines for management and follow-up.

As a relatively new entity, NCGS involves several unanswered clinical and research questions. In fact, it remains debatable whether the condition is even caused by gluten or, at least partially, by other components found in cereals [3–5]. There could also be substantial clinical overlap with irritable bowel syndrome (IBS) subtypes [3, 6]. Other currently poorly defined issues are the frequency of chronic co-morbidities in NCGS, health-related quality of life, and long-term prognosis of affected individuals. Interestingly, there is some evidence suggesting that individuals with NCGS may present with persistent symptoms and impaired well-being despite gluten avoidance [7]. Overall, however, the natural history and long-term treatment outcomes of NCGS have been little studied [8, 9].

We explored long-term outcomes of self-reported intolerance of gluten-containing cereals by inviting individuals undergoing thorough investigations due to this condition in the period 1995–1997 to a follow-up study. Emphasis was placed on persistence of symptoms, the possible development of CeD during follow-up, current health-related quality of life, and how many were still avoiding gluten-containing cereals after 25 years.

## Materials and methods

### Patients and study design

**Fig. 1:**
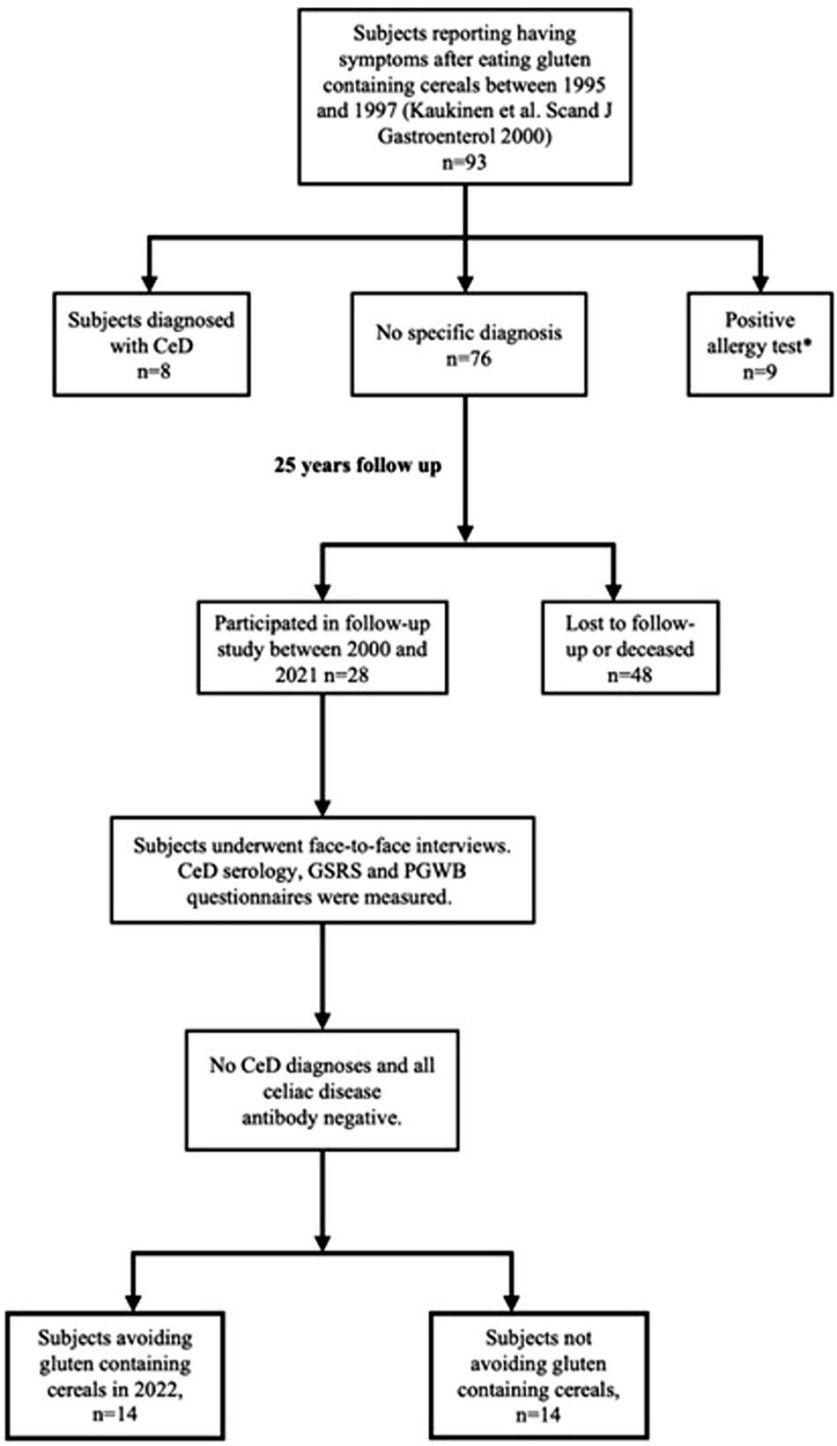
Flowchart of the study participants. * Specific and total IgE antibodies for wheat were measured with additional prick and patch testing. After testing, the dermatologist deemed nine patients as having possible cereal allergies and started an open-label elimination diet for them [9]. CeD celiac disease.

The study was conducted at Tampere University and Tampere University Hospital in 2021. The present study was a follow-up to one carried out in the same center approximately 25 years earlier [10]. The previous study included 93 consecutive adults who experienced gastrointestinal symptoms after consuming gluten-containing cereal products such as wheat, rye, and barley [10]. In the study conducted in 1995–97 [10], participants underwent a variety of diagnostic investigations at baseline, including upper gastrointestinal endoscopy with duodenal biopsies, determination of CeD-associated genetics and serology, and comprehensive allergy testing (Fig. 1). Altogether 76 participants did not have CeD or wheat allergy and, according to standard treatment protocols at that time, they were advised to revert to normal gluten-containing diet. Those individuals whose contact details were available in the digital and population data services agency of Finland were invited to a follow-up study. Participants were invited to take part through up to two letters, in accordance with ethical approval. Those who responded by contacting the study nurse were subsequently scheduled for a visit at the Tampere Celiac Disease Research Center.

During the study visit, participants were interviewed personally by a study nurse, and blood samples were taken for CeD antibody and laboratory measurements. The participants also completed specific questionnaires eliciting gastrointestinal symptoms and quality of life. The control groups comprised 128 untreated CeD patients collected from a prospectively maintained research database and 160 apparently healthy non-CeD individuals who were not on a gluten-free diet (GFD) and had no first-degree relatives with CeD [11]. The control patients were recruited through newspaper advertisements and via local and national CeD associations from different parts of Finland. “Untreated CeD” refers to individuals who had received a CeD diagnosis but were studied prior to diagnostic investigations and before starting a GFD. Following small-bowel mucosal biopsy findings of villous atrophy and crypt hyperplasia, these patients were placed on a GFD.

The study protocol and participant recruitment were approved by the Ethics Committee of the Pirkanmaa Hospital District. Written informed consent was obtained from all participants. The Declaration of Helsinki was followed throughout the study process.

### Clinical data

The following demographic and clinical data were collected from all participants: Age, self-reported gender, body mass index, possible CeD diagnosis or other chronic disease after the first study in 1995–97, and presence and duration of possible persistent gastrointestinal symptoms. In addition, the possible avoidance of gluten-containing cereals was systematically assessed using a written questionnaire, with the responses reviewed by a study nurse. Participants were asked whether their reason for gluten avoidance was symptoms, assumed negative long-term health effects, or other factors, with the option to select multiple reasons. Adherence to a possible GFD was further categorized as strict, occasional lapses, or no diet. Participants were also asked to rate the ease of following the possible diet on a scale of 1–5, its impact on daily life (yes/no), the frequency of follow-up (never, occasionally, every 2–3 years, yearly), and the current follow-up method (doctor, nurse, remote contacts, or other). The gastrointestinal symptoms were systematically assessed to evaluate potential fulfillment of the Rome IV criteria for IBS [12].

### Laboratory measurements

In this follow-up study, serum IgA-class tissue transglutaminase antibodies (TGA) were analyzed at the Tampere Celiac Disease Research Center by commercial assay (Celikey®, Phadia GmbH, Freiburg, Germany). Serum IgA-class endomysial antibody (EmA) titers were determined by indirect immunofluorescence using human umbilical cord as substrate [13]. In case of suspected IgA deficiency, the antibodies were measured in the IgG class. A serum dilution of 1 ≥ 5 for EmA was considered positive, and positive samples were further diluted up to 1:4000. Dynal SSP low-resolution DQ typing kit (Dynal AS, Oslo, Norway) was used to determine the presence of CeD-associated HLA DQ2 and DQ8 haplotypes [14]. Blood hemoglobin was measured with a commercial HemoCue Hb 201+ analyzer (Triolab Inc., Turku, Finland). Anemic values were defined based on sex-specific reference ranges: 117–155 in women and 134–167 in men [15].

### Questionnaires

Gastrointestinal symptoms and health-related quality of life and well-being were assessed quantitatively using three validated and widely used questionnaires, including the Gastrointestinal Symptom Rating Scale (GSRS), the Psychological General Well-Being Index (PGWB), and the Rand 36-Item Short Form Health Survey (SF-36). GSRS and PGWB were available from both participants and controls, while scores from a separate Finnish population sample (*n* = 2060, 1133 women and 927 men) were used for comparison of gender-specific SF-36 values. Altogether 1529 of these were aged 18–64 years, and 513 were over 65 years old [16].

GSRS estimates self-experienced gastrointestinal symptoms with 15 separate questions utilizing a Likert scale from 1 to 7 points, higher scores denoting more severe symptoms [17, 18]. Independent questions are further combined into subcategories of diarrhea, indigestion, constipation, pain, and reflux. The results are given as mean scores for total and subdimension scores.

The PGWB questionnaire assesses psychological health and well-being with 22 separate items, which are further combined into subcategories of anxiety, depression, well-being, self-control, general health, and vitality [17, 19]. Each question is answered on a Likert-like scale from 1 to 6, and the results are summed for total and subdimension scores. Higher scores indicate better self-perceived health and well-being [17, 19].

The SF-36 evaluates various aspects of health and quality of life [20]. It comprises eight subcategories, including physical functioning, bodily pain, role limitations due to physical health problems, role limitations due to personal or emotional problems, emotional well-being, social functioning, energy/fatigue, and general health perceptions. The scores of each item may range from 0 to 100 points, with higher values indicating better health status.

### Statistical analysis

Statistical analyses were conducted using IBM SPSS Statistics software (IBM Corp., Armonk, New York, USA). The results are given either as numbers of cases and percentages or as means with 95% confidence intervals (CI) or ranges. Visual inspection and Shapiro–Wilk and Kolmogorov–Smirnov tests were used to confirm the normality of the data. Independent samples *T*-test, Mann–Whitney *U* test, and chi-square were used for testing statistical significance when appropriate. In addition, a one-sample T-test was used when comparing the SF-36 scores of the study participants to the scores of the Finnish population sample. A two-tailed *p*-value < 0.05 was considered significant in all comparisons. A separate comparison analysis was conducted between those ingesting gluten-containing products and those avoiding them.

## Results

### Clinical characteristics and laboratory evaluation

Altogether, 28 individuals with abdominal symptoms after the ingestion of gluten-containing cereals participated in the present follow-up study, achieving a follow-up rate of approximately 40%, which can be considered fair given the exceptional length of the study [21] (Fig. 1). Most of them, as well as individuals in the two control groups, were women, and mean age and body mass index were higher among participants than among controls (Table 1). In total, 61% of the participants reported allergy, 39% hypertension, 28% asthma, 25% hypothyroidism, 21% rheumatic disease, 21% osteoporosis, 21% depression, 14% had type 2 diabetes, and 14% malignancy. Allergies include hypersensitivity to pollen, household pets, and antibiotics, and to various foods, including grains (wheat allergy excluded), milk, fruit, chocolate, and soybeans. No comparable data on chronic diseases were available from the controls.

**Table 1:** Demographic and clinical data of the study participants and controls.

	Participants			Controls	
	Total *n* = 28	Avoiding gluten-containing cereals *n* = 14	Ingesting gluten-containing cereals *n* = 14	Healthy controls^a^ *n* = 160	Untreated CeD *n* = 128
Females, *n* (%)	20 (71.4)	13 (92.9)^*^	7 (50.0)^*^	115 (71.9)	97 (75.8)
Age, mean (range), years	62 (43–79)^*^	64 (46–75)	61 (43–79)	55 (23–87)^*^	47 (15–72)^*^
BMI, mean (range), kg/m^2^	27.1 (19.2–56.1)^*^	25.9 (19.2–31.9)	28.3 (20.6–56.1)	NA	25.1 (16.6–39.1)^*^
CeD diagnosis, *n* (%)	0	0	0	0	128 (100)
Positive CeD serology^c^, *n* (%)	0 (0)	0 (0)	0 (0)	0 (0)	128 (100)
HLA DQ2 or DQ8, *n* (%)	14/24 (58.3)^*^	8/12 (66.7)	7/12 (58.3)	NA	94/94 (100)^*^
Fulfills IBS criteria^b^, *n* (%)	12 (36.1)	5 (35.7)	7 (50.0)	NA	NA
Avoiding gluten-containing cereal(s), *n* (%)	14 (50.0)	14 (100)	0	0	0
Diet adherence, *n* (%)					
Strictly avoiding cereals	7 (25.0)	7 (50.0)	0	0	0
Occasional lapses	7 (25.0)	7 (50.0)	0	0	0
No diet	0 (0)	0 (0)	14 (100)	160 (100)	128 (100)

*NA* not available, *BMI* body mass index, *CeD* celiac disease, *IBS* irritable bowel syndrome.

^*^p < 0.05, *t*-test or chi-square applied as appropriate.

^a^No first-degree relatives with CeD.

^b^According to the Rome IV criteria.

^c^Serum IgA-class tissue transglutaminase and endomysial antibodies.

All participants tested negative for TGA and EmA in the current evaluation. Samples for genetic analyses were obtained from 24 participants, of whom 14 had HLA DQ2/DQ8 and 10 (42%) other HLA types (Table 1). Hemoglobin results were available from 26 participants, of whom 24 had normal values, while one woman (113 g/l) and one man (124 g/l) had borderline anemic values. None of the 28 participants had developed CeD or acquired a diagnosis of any other gastrointestinal disease during follow-up. In a separate analysis, half of the participants (50%) were actively avoiding gluten-containing cereals at the follow-up, with this practice being more common among women (*p* < 0.05) compared to those consuming a gluten-containing diet (Table 1). No other significant differences were observed between the subgroups.

### Gastrointestinal symptoms and quality of life

Since the study participants were older than the controls and all >40 years of age (Table 1), only controls >40 years of age were included for comparison of gastrointestinal symptoms and quality of life [22]. This resulted in 82 untreated CeD patients and 130 healthy controls for GSRS comparisons and in 68 patients and 77 controls, respectively, for PGWB comparisons.

**Fig. 2:**
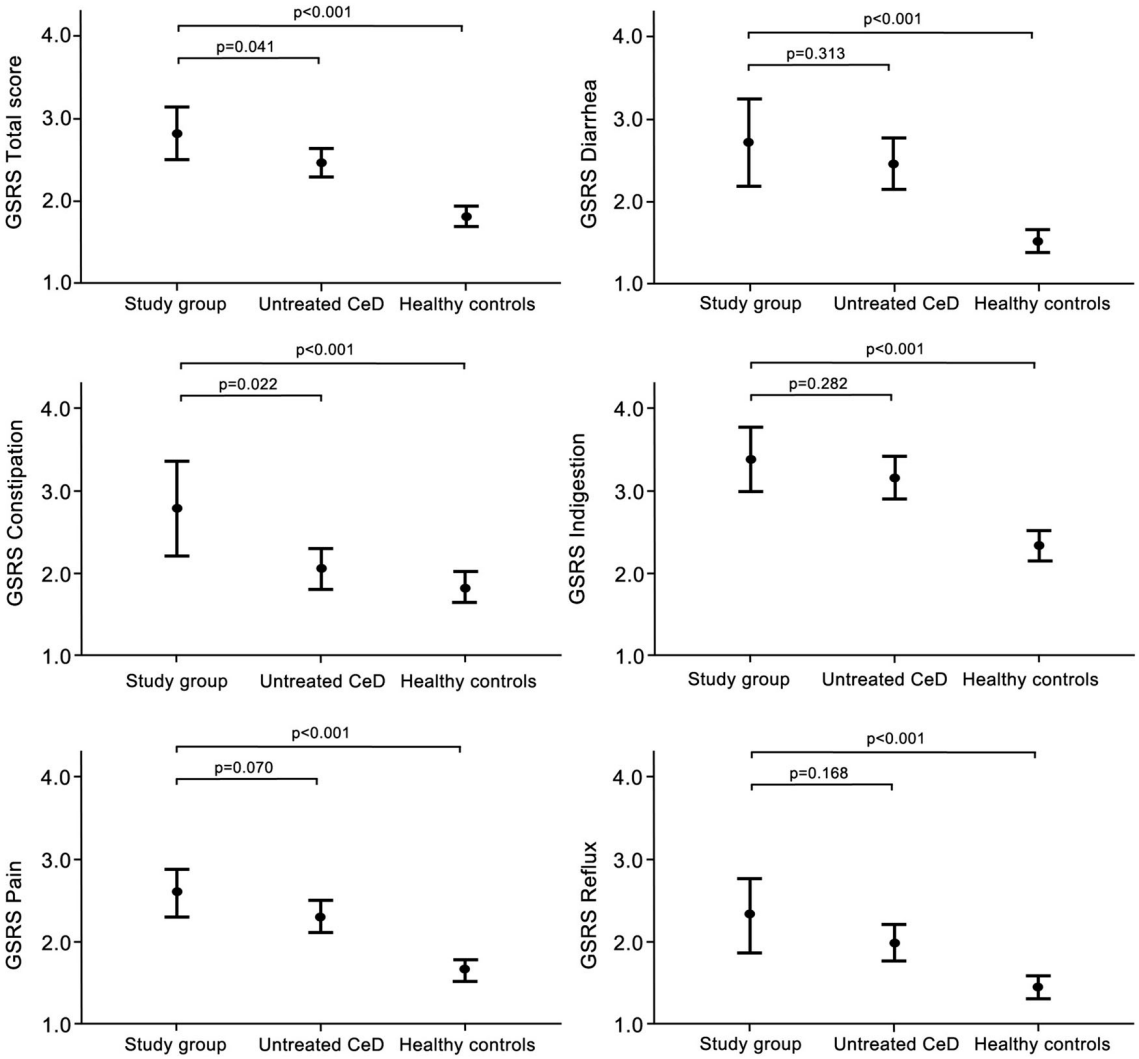
Gastrointestinal Symptom Rating Scale (GSRS) total and subdimension scores in individuals avoiding gluten-containing cereals after 25 years (*n* = 28), compared to untreated celiac disease (CeD) patients (*n* = 82) and healthy controls (*n* = 130). The results are shown as means with 95% confidence intervals. Higher GSRS scores denote more severe gastrointestinal symptoms.

Study participants reported more severe gastrointestinal symptoms, as indicated by higher GSRS total scores and all sub-scores compared to healthy controls, as well as higher total and constipation scores than untreated CeD patients (Fig. 2). There were no significant differences in GSRS sub-scores for pain, diarrhea, indigestion, and reflux between study participants and healthy controls or untreated CeD patients (Fig. 2).

**Fig. 3:**
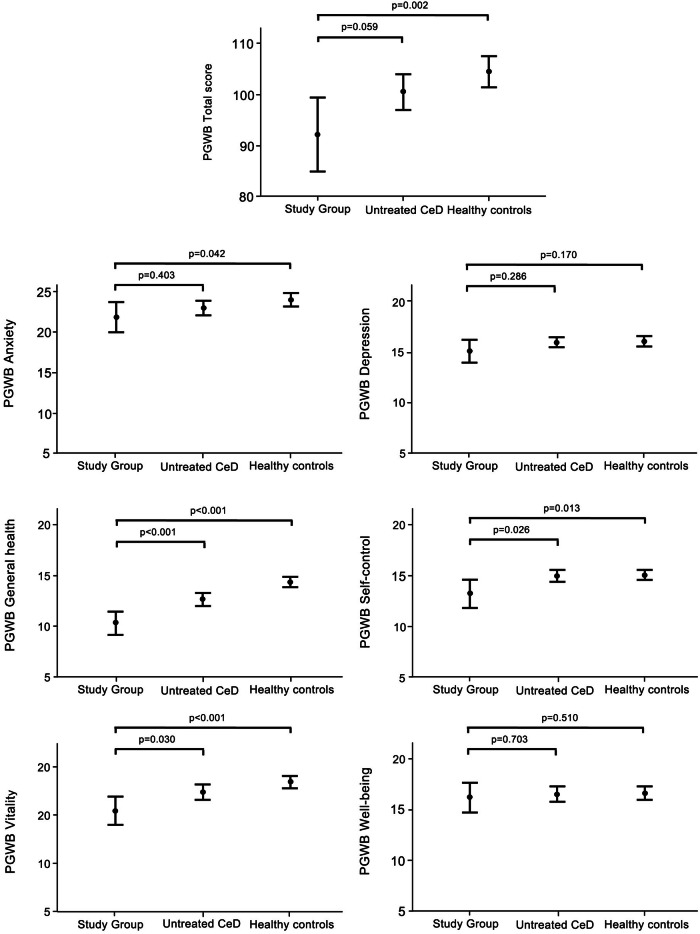
Psychological General Well-being (PGWB) total and subdimension scores in individuals avoiding gluten-containing cereals after 25 years (*n* = 28), compared to untreated celiac disease (CeD) patients (*n* = 68) and healthy controls (*n* = 77). The results are shown as means with 95% confidence intervals. Higher PGWB scores denote better well-being.

The participants also had poorer PGWB total and anxiety, self-control, general health, and vitality scores than the healthy controls, and poorer self-control, general health and vitality scores than untreated CeD patients (Fig. 3). On the SF-36, both women and men participants had worse mean general health scores (women 51.8 [95% CI 39.8–63.7] vs. 65.4 respectively, *p* = 0.028; men 44.4 [27.6–61.2] vs. 64.4 respectively, *p* = 0.026) and bodily pain scores (women 56.3 [46.0–66.5] vs. 61.5, *p* = 0.001; men 56.6 [41.7–71.5] vs. 77.6, *p* = 0.012) than the population sample. There were no significant differences in other SF-36 subdimensions (data not shown).

No significant differences were observed in GSRS, PGWB, and SF-36 scores in subgroup analysis between those avoiding and those not avoiding gluten-containing cereals (data not shown). The reason for avoiding gluten-containing cereals was due to experienced symptoms in twelve participants and concerns about long-term disadvantages in two. The sub-question “ease of following the diet” in these participants had a mean score of 2.42 on a scale of 1–5, with 6 out of 14 reporting that the dietary restriction affected their daily life. Seven out of 14 participants were followed up due to cereal avoidance; however, follow-ups were scarce, with only one participant receiving regular annual follow-ups and six being monitored occasionally (less than every 2–3 years). Of these seven followed individuals, four reported regular doctor visits, while three had remote doctor consultations (e.g., telephone calls) with prior laboratory assessments.

## Discussion

None of the participants in the present study who originally reported symptoms from gluten-containing cereals developed CeD or had acquired any diagnosis of an intestinal disease during the 25-year follow-up. This is somewhat in contrast to earlier studies, which have reported undiagnosed CeD in some individuals with NCGS [23]. It must be emphasized that our participants had already undergone meticulous exclusion of CeD in the original study [10]. A specific gluten challenge was particularly important in the baseline study, as an already initiated GFD could result in a false negative CeD diagnosis [10, 24]. It is, however, important to bear in mind that half of the participants were still avoiding gluten-containing cereals, which may mask possible CeD at this point. Furthermore, not all participants in the original study were able to maintain a gluten-containing diet for more than 2–3 weeks before the diagnostic studies [10]. Interestingly, new diagnostic tests for CeD are being developed to allow for diagnosis without the need for the laborious gluten challenge [25, 26]. However, these tests have not yet been implemented in clinical practice. On the other hand, diagnostic methods for NCGS are also being explored. Approaches such as anti-gliadin antibodies and zonulin measurement show promise, but their diagnostic accuracy remains currently insufficient for routine clinical use [27, 28].

Comparable long-term studies are lacking, but Carroccio et al. [9] followed 200 non-CeD wheat-sensitive (NCWS) individuals for a median of 99 months. At the end of the study, 74% of them were still on a strict wheat-free diet, and 90% reported alleviation of symptoms [9]. Here, there was no significant difference in the gastrointestinal symptoms or quality of life scores of participants currently avoiding and not avoiding gluten-containing cereals, but the subgroups were likely too small for reliable comparisons. Then again, although half of the participants adhered to a dietary restriction, the entire study group still displayed more gastrointestinal symptoms and poorer quality of life than controls or even untreated CeD patients, and worse SF-36 general health and pain scores than the general population. In line with this, Tovoli et al. found individuals with NCWS to have more symptoms than CeD patients after one year on a GFD [29], and Skodje et al. found NCGS patients to suffer from various gastrointestinal and extraintestinal symptoms and impaired quality of life despite a concomitant GFD and low or moderate intake of fermentable oligosaccharides, disaccharides, monosaccharides, and polyols (FODMAP) diet [7]. Of note, almost half of the participants in this study fulfilled the Rome IV criteria for IBS currently without prior diagnosis of the disease. Biesiekierski et al. reported that NCGS patients with IBS experienced alleviation of symptoms on a low FODMAP diet [4], and Skodje et al. [3] found that fructan induced more symptoms in NCGS patients than gluten during a double-blind challenge. As a low FODMAP diet involves reduced gluten intake, it is possible that IBS and NCGS are at least partly variants of the same entity [30].

Regarding genetic background, 63% of the participants were HLA DQ8/DQ2 positive, which is more than reported in the general population (30–40%) and individuals with NCGS (50%) [14, 31]. It has been postulated that these haplotypes could predispose not only to CeD but also to NCGS [28], but more evidence is needed. An additional suggested cause of the clinical manifestations – at least in some NCGS patients – is non-IgE-mediated food allergy [32]. Notably, up to 61% of the participants in this study reported some allergy, although they had negative wheat allergy testing at baseline. Taken together, there may be several mechanisms behind clinical intolerance of gluten-containing cereals, and in many cases, gluten may actually not be the main culprit [33].

Besides impaired quality of life, total and many sub-scores, depression was three times more common among the study participants than has been previously reported in the general Finnish population [34]. Depression is a well-known characteristic of untreated CeD, whereas the association between depressive symptoms and NCGS has been scarcely studied [35]. Supporting a direct causal link, Peters et al. [36] reported that even a short-term exposure to gluten resulted in elevated depression scores compared to placebo in individuals with NCGS, despite no simultaneous increase in gastrointestinal symptoms. More studies on this issue are needed, but it appears that individuals with NCGS often face a high psychological burden and thus need intensified surveillance and specific support from healthcare. Interestingly, half of those currently avoiding gluten-containing cereals reported to be followed due to their diet; however, follow-ups were infrequent, with intervals of at least several years between contacts. Of note, all follow-ups were conducted with doctors, and none with dietitians.

The main strengths of the present study are the unique cohort of participants who had undergone exclusion of CeD and other gastrointestinal diseases in the original study in the 1990s, which was before gluten sensitivity became a recognized phenomenon in the 2010s. Additionally, there was comprehensive testing for CeD serology and genetics, and current symptoms and quality of life were evaluated by validated and widely used questionnaires, with representative control groups. As a limitation, the attendance rate was moderate, around 40%. This figure is generally considered acceptable in studies spanning months or a few years [21]; however, given the exceptional length of the follow-up, it could even be considered commendable. An additional potential limitation is the risk of unrecognized CeD in some of the controls. Furthermore, the exclusion of gastrointestinal diseases other than CeD in the follow-up study was conducted solely through interviews and by reviewing the participants’ medical history. However, all of them had undergone thorough screening for gastrointestinal diseases at baseline, which included a gastroenterology consultation with duodenal biopsies and an allergist evaluation with allergy testing.

To conclude, the development of CeD or the acquisition of a diagnosis of any other gastrointestinal disease in individuals with intolerance to gluten-containing cereals appears to be rare, even during long-term follow-up. However, these individuals reported more gastrointestinal symptoms and a poorer quality of life than the general population or even untreated CeD patients. Moreover, up to 50% continued to avoid gluten-containing cereals even 25 years after the original study ended. Thus, among individuals without CeD avoiding gluten consumption, there seems to be a need for individualized nutritional guidance and psychological support.

## Data Availability

The datasets generated during and/or analyzed during the current study are available from the corresponding author on reasonable request.
